# Supplementary material for: Are carbon emissions trading and green financial instruments synergistic? -Comprehensive quantitative research based on content analysis
Source: PLoS One. 2024 Mar 7;19(3):e0298601. doi: 10.1371/journal.pone.0298601 (PMC10919601; doi:10.1371/journal.pone.0298601)
Supplement: S1 Data — (ZIP) [file pone.0298601.s001.zip › Data/地级市Z_F工作报告.zip!/╦─┤¿/│╔╢╝╩╨/└┤╫╘╣1⁄2╓┌║┼í╛┬φ┐╦╩2╛▌═°í┐_2014.docx]

各位代表：

我代表市人民政府，向大会报告工作，请予审议，并请市政协委员提出意见。

2013年工作回顾

2013年是我市全面贯彻党的十八大精神的重要一年。在党中央国务院、省委省政府和市委正确领导下，在市人大市政协监督支持下，市政府围绕打造西部经济核心增长极，深入推进五大兴市战略，积极应对宏观经济下行压力、“4•20”芦山强烈地震和汛期暴雨洪涝灾害带来的不利影响，全市经济社会继续保持良好发展势头。预计全年地区生产总值迈上9000亿元台阶，增长10%以上；地方公共财政收入898.5亿元，增长16.6%；固定资产投资6500亿元，增长10.4%；城镇居民人均可支配收入29913元、农民人均纯收入12880元，分别增长10%和12%；城镇登记失业率2.82%；万元地区生产总值能耗下降3.2%。获批首批全国质量强市示范城市和国家公共文化服务体系示范区，成功举办2013《财富》全球论坛和第十二届世界华商大会。

一、经济发展实现稳中快进

一是工业经济快速增长。预计实现规模以上工业增加值2800亿元，增长13.4%。重大项目加快建设，完成工业投资1700亿元，一汽大众三期扩能等159个项目竣工投产，捷普基地、新筑轨道交通等266个项目进展顺利。八大特色优势产业快速发展，其中汽车、电子信息产业分别增长40%、15%以上，新一代信息技术、生物医药、节能环保等战略性新兴产业占全市工业比重提高1.5个百分点。深入实施大企业大集团培育工程、中小企业成长工程和微型企业扶持计划，新增百亿企业2户，新登记企业4.6万户。全面推行企业“首席质量官”制度，新增中国驰名商标7件。依法关停和淘汰落后产能企业88户，单位工业增加值能耗下降10%以上。

二是现代服务业加快发展。预计实现服务业增加值4520亿元，增长8.5%。服务业重大项目完成投资1086.6亿元，社会消费品零售总额3730亿元，增长12.6%。总部经济等高端服务业加速发展，连锁经营、同城配送等新型商贸业态发展迅速，电子商务交易额突破4000亿元。金融总部商务区和成都民间金融街建设进展顺利，银行机构、保险机构分别增至61家、72家，总量继续保持中西部第一。新开通至法兰克福、伦敦等4条国际直飞客运航线及14天直达波兰罗兹的货运“蓉欧快铁”，荣获2013中国物流中心城市杰出成就奖。全市接待旅游总人数1.55亿人次，实现旅游总收入1330亿元，同比均增长26%，被评为2013中国最佳休闲城市。成功举办全国糖酒会等会展节庆活动，会展业实现综合收入510亿元，增长21%，荣获2013中国十佳品牌会展城市称号。

三是现代农业较快发展。预计实现农业增加值320亿元，增长3.5%，农业产业化重大项目完成投资90.5亿元。产业结构不断优化，制定实施农产品加工业布局规划，现代种业、绿色有机农业和农产品精深加工业加快发展，农产品精深加工率达42%。创新“米袋子”和“菜篮子”保障机制，实施水稻规模化生产奖励政策，在全国副省级城市率先推出政策性蔬菜价格指数保险。产业化经营成效明显，新发展农民合作社561家，新培育农业职业经理人2500人、新型职业农民2.1万人，家庭农场增至833家。农田水利设施不断提升，东风水库扩建完成主体工程，龙泉山“百湖”水源工程加快建设，新增节水灌面21.9万亩。

二、对外开放合作成效显著

一是成功举办《财富》全球论坛和世界华商大会。2013年6月6日和9月24日，《财富》全球论坛和第十二届世界华商大会分别在我市举办，习近平总书记发来贺信，李克强总理在京会见出席财富论坛的企业家代表，俞正声主席和张高丽副总理分别出席华商大会和财富论坛并作主旨演讲。英国前首相布莱尔等5名外国前政要、622名嘉宾以及300余家世界500强企业和国际知名企业的代表出席财富论坛，105个国家（地区）3000多名代表参加华商大会，现场共签约重大项目121个、投资总额1413.5亿元。超过200家境内外媒体来蓉采访报道，有力推动了让世界了解成都、让成都走向世界，论坛后续效应不断显现。

二是投资促进成效明显。预计实际到位内资3300亿元，实际利用外资103.1亿美元，分别增长33.2%和20%。组织开展系列主题投资促进活动，新引进法国苏伊士、荷兰全球保险等世界500强企业22家，在蓉世界500强企业增至252家。加强重大产业化项目市域统筹，高新区、经开区、天府新区等重点区域招商取得明显进展，新签约德州仪器芯片封装测试、联想区域结算中心、益海嘉里等重大项目400个。进一步优化外商投资企业和民营企业诉求解决机制，及时帮助企业解决生产经营中遇到的困难和问题。

三是外经外事提升拓展。预计实现进出口总额490亿美元，增长3.1%，其中成都高新综保区实现进出口253亿美元。进一步优化外贸商品结构和出口市场，以便携式电脑为主导的机电产品出口大幅提升，东盟、南亚、俄罗斯等新兴市场出口快速增长，维纳软件等5家企业成为中国服务外包成长型企业100强，地奥、康弘公司的自主研发药品获批进入欧美市场。澳大利亚驻蓉领馆正式开馆，新增印度班加罗尔等4个友好城市，与德国波恩建立中欧城镇化合作示范城市伙伴关系。成为继北京、上海、广州之后全国第四个获批72小时过境免签政策城市。

四是区域合作不断深化。成都经济区扩容为包括阿坝州、甘孜州、凉山州在内的区域合作。编制完成《成德绵一体化总体规划》，签署《成都德阳同城化发展框架协议》，以中江县为合作支点共建成德工业园区。成资、成眉等共建工业园区完成投资67.2亿元、引进项目40个。建立成都经济区秸秆综合利用区域合作机制和医疗协查结果通报机制。与长三角、珠三角、环渤海城市合作全面加强。对口援藏工作扎实开展，启动实施对口援建项目70个，完成投资1.6亿元。

三、城市建设管理加快提升

一是交通通信枢纽加快建设。着力提升西部综合交通主枢纽功能，成都新机场完成选址，双流机场年旅客吞吐量突破3300万人次，位列全国城市第四位，成都铁路调度指挥所、成灌快铁离堆支线建成投用，成绵乐客专、成蒲铁路等加快建设。不断完善市域交通体系，第二绕城高速主体工程完工，大件路外绕线、新邛路等市域快速通道加快建设。强力推进中心城区缓堵保畅工程，二环路高架、BRT快速公交及智能交通管控系统建成运行，地铁2号线西延线开通运营，4号线等5条地铁建设加快推进。继续提高中心城区公交出行分担率，新增公交车2000辆，在全国率先开通运行免费社区巴士60条，实行早高峰错时公交免费刷卡政策，BRT快速公交和地铁日均载客达90万人次。“光网城市”建设提速，建成4G基站2800个，被工业和信息化部批准为国家级互联网骨干直联点城市。

二是天府新区建设全面提速。天府新区成都管委会正式成立并全面运行。编制完成成都片区控制性详细规划，基本完成重要道路、重点区域和重要节点城市设计。天府大道南延线、元华路南延线等“三纵一横”二期基本形成通车能力，天府大道三期开工建设，重要节点立交改造加快实施，华阳东、黄水220千伏输变电站建成投用，天然气管线和给排水主干网及配套建设全面开工，完成固定资产投资274亿元。新川创新科技园、龙泉国际汽车城、双流电子信息产业园、新津新材料产业园等功能区加快建设，新签约引进重大项目58个。兴隆湖、锦江生态带等重点生态工程加快推进。

三是城市更新改造明显加快。北改工程深入实施，累计启动项目409个，完成投资557亿元，曹家巷、八里庄二仙桥、昭觉寺等片区改造顺利，川陕路、熊猫大道等11条主干道路建成通车，荷花池市场等传统市场完成拆除。锦江区、成华区、青羊区和武侯区“三无”院落完成整治和实现自治，南光厂宿舍等棚户区加快改造，宽堂窄门等历史文化片区整治提升，二环路、人民南路等6条主干道完成立面整治。建成停车场等公建配套项目379个，新增停车泊位3.5万个。启动小城市和特色镇建设，一般场镇改造完成项目377个、投资45.3亿元。

四是城乡环境治理深入推进。深化市容环境综合治理，强化环境卫生、市容秩序、道桥病害、集贸市场等突出问题整治，市容市貌明显提升。深化大气环境综合治理，完成水泥等重点行业企业节能减排设施建设，中心城区全面供应国Ⅳ标准燃油，实施建筑工地扬尘、秸秆焚烧等专项治理，实现国控污染源二氧化硫在线监控安装率100%。深化水环境综合治理，启动中小河流整治三年行动计划，完善乡镇污水处理厂配套设施，完成中心城区1792个下河排水口和30条黑臭河渠治理，被水利部评为全国水生态文明建设试点城市。深化城乡生态环境建设，基本形成绕城高速200米生态带，锦城湖、白鹭湾湿地、锦江滨河公园一期建成开放，新增健康绿道92公里、城市绿化65万平方米，森林覆盖率达37.8%。全国环境保护模范城市复核验收顺利通过。

四、综合配套改革不断深化

一是农村市场化改革积极推进。巩固农村产权改革成果，深入推进农村产权管理体系和集体资产量化确权工作，累计发放耕保金61.1亿元。稳步推进农村生产要素规范有序流动，农村产权交易所交易建设用地指标657宗，交易金额132亿元，农村产权抵押融资余额超过60亿元。实施统筹城乡示范镇综合改革，推进农民自主开发集体建设用地等改革试点。全面开展农用地“转征与实施分离”审批改革试点，鼓励农民自主实施土地综合整治，完成农用地整理66.3万亩。

二是农村公共服务深化拓展。加快农村公共服务和社会管理设施标准化建设，在全国率先制定涉农社区和村公共设施配置标准体系。村级公共服务和社会管理专项资金上调至每村每年40万元，新启动融资项目73个。开展乡镇重大公共建设项目民主管理改革试点，实施小型公共基础设施村民自建改革项目156个。推进农村道路联网加密和提档升级，实施农村居民饮水保障提升工程，村和涉农社区自来水覆盖率达80%。

三是社会管理改革切实加强。深化城乡统一户籍制度改革，建立公民信息管理系统，推动流动人口服务管理向实有人口服务管理转变，基本实现进城务工人员与城镇居民享受同等的子女教育、卫生计生等公共服务。深化社会组织管理改革，完善社会组织分类发展管理办法，拓展社会组织直接登记范围，社工专业人才增长26%。理顺地名标牌管理机制，加强地名规范化管理。完善街道管理和村社治理机制，全面推进居务公开，推广社区网格化管理，在全国首创社区公共财政制度，村民议事会能力和水平进一步提升。

五、城乡社会事业全面进步

一是就业社保稳步提高。实施民生工程163项，财政投入资金412亿元。深入开展就业行动计划，积极促进高校毕业生等重点人群就业创业，城镇新增就业24.6万人，农村劳动力新增转移就业10.6万人，城乡失业人员再就业9.7万人，动态消除“零就业”家庭。完善城乡社会保障全覆盖体系，在全国率先建立城乡居民养老保险待遇正常调整机制，实施基本医疗保险金预算管理和总额控制，符合条件低保对象4676人整体纳入城镇职工养老保险，全市提高低保标准10%以上并实现同一区（市）县内城乡统一，建立社区快速救助机制，被人力资源和社会保障部确定为全国劳动保障监察“两网化”管理示范城市。健全社会养老服务体系，投资1亿元建成6家公建民营养老机构，新增民办养老机构和社区居家养老服务中心153家，新筹建养老床位1.2万张。发放住房公积金贷款130亿元，竣工各类保障性住房2.7万余套，实现公共住房保障应保尽保。第三轮第二批100个村的扶贫开发工作全面启动。

二是教育卫生加快发展。深入推进部省市共建统筹城乡教育综合改革试验区建设，公办和公益性幼儿园学位覆盖率达69.8%，幼儿入园率达98%，中小学校新一轮标准化提升工程加快推进，完成265所学校运动场改造，建成中小学数字化图书管理平台，实施惠及远郊22.7万名农村中小学生的营养改善计划，职业教育和特殊教育加快发展，成为全国首个整体实现县域义务教育基本均衡发展的城市。深化医药卫生体制改革，实施基层医疗卫生机构标准化建设设备提升工程，积极推进“医药分开”，36家县级公立医院取消药品加成，免费孕前优生健康检查扩面提质，被国家卫生和计划生育委员会评为全国创建幸福家庭活动试点单位和流动人口卫生计生基本公共服务均等化试点城市。

三是科技文化不断进步。深入推进创新型城市建设，完善科技创新和知识产权保护政策，支持企业牵头建设研发机构，实施产学研合作项目800余个，新增科技创新载体34家、孵化面积61万平方米，新增高新技术企业312家，全市发明专利申请量增长33.7%，成为国家首批智慧城市试点示范城市。加快“文化之都”建设，设立公共文化服务专项资金和政府采购资金，建成农村标准化广播室471个、农家书屋463个，新声剧场等重大文化项目加快建设，第四届中国成都国际非物质文化遗产节受到联合国教科文组织高度评价。组织开展“太极蓉城”等系列群众体育活动，被国家体育总局评为全国群众体育先进单位。地方志、参事、档案、哲学社会科学、科普等工作切实加强。

四是平安成都扎实推进。强化社会治安管理，加强城乡结合部、车站等复杂公共场所综合治安管理，集中整治“黄赌毒”等社会丑恶现象，严厉打击各类违法犯罪活动，加强特殊人群和虚拟社会管理，治安案件万人发案数下降8.2%，确保了《财富》全球论坛等大型活动圆满顺利。加强食品药品安全监管，积极推进食品药品监管体制改革，启动中药材流通追溯体系试点，肉类蔬菜溯源体系实现扩面提质，开展食品安全“百日会战”及中药材市场等专项整治。严格安全生产监管，深入开展安全生产大检查，强化道路交通、消防、危化品等重点行业领域专项整治，生产性安全事故起数下降14%左右。深入开展法治大讲堂活动，完善接访下访机制，强化矛盾纠纷排查化解，积极化解信访积案，全市信访总量下降13.1%。民族宗教、移民、统计、保密、侨台、人防、双拥等工作取得新成绩。

六、成功应对“4·20”芦山地震和汛期暴雨洪涝灾害

一是迅速开展抗震救灾。“4•20”芦山地震造成我市邻近震中的邛崃部分乡镇13万人受灾，4人遇难，农房倒塌1865间、严重损坏2.8万间。灾情发生后，迅速组织人员开展救援，派出医护人员救治转运伤员，调集救援物资运往受灾地区，确保了受灾群众得到及时救援和妥善安置，受灾地区社会安定有序。同时，积极支援雅安灾区抗震救灾，转移收治雅安地震伤员441人，定向为雅安灾区捐款捐物折合人民币4016.5万元。

二是积极应对暴雨洪涝灾害。7月汛期我市出现多次区域性特大暴雨并造成山洪泥石流灾害，全市90万人受灾，53人遇难，126人失踪，房屋倒塌1159户，地质灾害715处。灾情发生后，迅即开辟抢险救灾应急专用通道，调集救援人员开展救援，转移受灾群众12.6万人，房屋倒塌受灾群众全部得到安置，并妥善做好善后处置，确保了受灾县（市）社会稳定。

三是加快实施灾后重建。编制完成邛崃市“3+5”首批示范点规划，全面完成灾区重建选址和地质灾害评估，完成地质灾害隐患点排查4207处，实施地质灾害治理和应急排危工程36项。优先实施灾区住房重建，启动农房重建2449户，完成26224户农房维修加固，引导农民向集中居住区转移居住，确保受灾群众安全温暖过冬。同时，灾区水毁工程全面开展修复。

七、政府自身建设切实加强

一是党的群众路线教育实践活动扎实开展。按照中央、省委和市委开展党的群众路线教育实践活动统一部署，认真落实“照镜子、正衣冠、洗洗澡、治治病”的总要求，广泛听取各方面意见建议，深入查找“四风”方面突出问题，组织召开专题民主生活会，开展批评与自我批评，并针对问题制定整改方案，以“钉钉子”的精神抓问题整改，确保应改必改、应改尽改。进一步完善惩治和预防腐败体系，加强领导干部任期经济责任审计，深入开展社保、教育、医疗等九大维护群众权益专项行动。严格“三公”经费管理，强化“文山会海”和节庆论坛等治理，市政府下发文件和全市性会议分别减少18.6%、12.2%，公务接待费和因公出国（境）费分别减少51.4%、34.4%。

二是行政效能不断提升。深化行政审批制度改革，启动商事登记制度改革，精简调整下放79项行政审批项目，取消和停收30项行政事业性收费，实现市县两级协同联动审批。深化政务公开，健全行政权力公开运行机制，清理完善公共企事业单位办事公开名录，主动公开信息14.7万余条。严格国有资产监管，建立公共资源交易中心，基本完成事业单位改革前期工作。加强电子政务建设，启动移动网上政务大厅建设，90.6%的行政审批和服务事项实现网上审批。连续三年荣获中国服务型政府十佳城市称号。

三是依法行政深入推进。严格执行市人大及其常委会的各项决议决定，自觉接受市人大及其常委会法律监督、工作监督和市政协的民主监督，认真做好人大代表和政协委员的建议、提案工作，累计办结建议、提案1267件，办复率100%，人大代表、政协委员满意和基本满意率99.6%以上。全面推进法治政府建设，完善科学民主依法决策制度，开展“规范行政执法年”活动，自觉接受社会监督和新闻舆论监督。深入实施“六五”普法，健全城乡一体的法律服务体系。

各位代表，过去一年成绩来之不易。这是党中央国务院亲切关怀的结果，是省委省政府和市委正确领导的结果，是市人大市政协和社会各界支持监督的结果，是全市人民共同努力的结果。在此，我代表市人民政府，向在各个领域和岗位上辛勤劳动、作出贡献的全市人民，向给予我们支持与监督的人大代表、政协委员、各民主党派、工商联和无党派人士、各人民团体、社会各界人士，向大力支持我们工作的中央和省驻蓉单位、解放军和武警驻蓉部队，向关心支持成都建设发展的港澳台同胞、海外华人华侨和国际友人，表示衷心感谢并致以崇高敬意！

在看到成绩的同时，我们也清醒看到，当前我市经济社会发展还存在不少问题和薄弱环节：转变经济发展方式、优化调整产业结构力度有待进一步加大，统筹城乡发展等重点领域改革有待进一步突破，城市交通拥堵、环境污染等“城市病”问题治理有待进一步加强，就业增收、养老社保、学前教育等民生工程有待进一步提升。此外，需要特别说明的是，由于受国内外宏观经济下行压力、“4•20”芦山地震、汛期暴雨洪涝灾害，以及我国经济从高速增长转向中高速增长换档期的影响，虽然预计全年地区生产总值增速高于全国2.4个百分点，但是距年初的预期目标相差2个百分点，同时城乡居民人均收入也比预期分别相差3.5和2.5个百分点。对此，我们将在今后工作中加倍努力，积极解决。

2014年工作安排

2014年是贯彻落实十八届三中全会精神、全面深化改革的开局之年，也是奋力打造西部经济核心增长极、加快建设现代化国际化大都市的攻坚之年。按照市委部署，做好今年政府工作要贯彻落实党的十八届三中全会、省委十届三次全会精神和中央、省委经济工作暨城镇化工作会议部署，把握“稳中求进、改革创新、统筹发展”的工作基调，坚持以全面深化改革为统领、以提高增长质量和效益为中心、以保障和改善民生为目的，深入推进经济社会转型升级，促进全市经济持续健康发展、社会和谐稳定。

2014年全市经济社会发展主要目标是：地区生产总值增长10%，固定资产投资总额6600亿元，地方公共财政收入同口径增长11%，城镇居民人均可支配收入增长10%，农民人均纯收入增长11%，城镇登记失业率控制在4%以内，万元地区生产总值能耗进一步降低，主要污染物排放量进一步减少。实现上述目标，要重点抓好八个方面工作。

一、加快改革创新，不断增强城市发展活力和动力

坚持市场在资源配置中的决定性作用和更好发挥政府作用，充分发挥全国统筹城乡综合配套改革试验区先行先试优势，敢于创新、勇于突破，加快重点领域和关键环节改革创新。

一是统筹城乡改革要有新突破。深化农村产权制度改革，探索农业生产用房等确权颁证，推进集体经济股份化，培育城乡统一的建设用地市场，赋予农民更多财产权利。推进城乡要素平等交换，健全农村产权交易所功能，完善城乡建设用地增减挂钩和耕地占补平衡指标交易制度，探索市域统筹使用建设用地指标，拓展农村产权抵押融资范围。加快农业经营体制创新，推进土地承包经营权、集体经营性建设用地使用权入股组建新型股份合作社，培育新型农业经营主体，发展多种形式的规模经营。深化城乡户籍制度改革，合理控制中心城区人口规模，引导鼓励农村人口向城镇自愿梯度有序转移，积极推进城镇基本公共服务常住人口全覆盖。

二是创新驱动发展要有新成效。深化科技体制改革，健全技术创新市场导向机制，发挥市场对技术研发方向、要素价格、各类创新要素配置的导向作用，加强创新链与产业链的对接协同，全社会研发投入增长15%以上。健全产学研协同创新机制，强化企业在创新中的主体地位，加强市场创新、管理创新和商业模式创新，推进企业技术进步和产业质量提升，实施100个重大科研项目，建设国家智慧城市试点示范城市，启动国家知识产权局专利审查协作中心建设。优化创新驱动发展环境，加大科技创新和成果转化政策支持，培育更多科技企业孵化器等创新载体，争取更多国家级研发平台布局，吸引更多高层次创新人才，推动更多科技成果惠及民生，努力营造良好创新创业社会氛围，着力打造全国一流的创新型城市。

三是对外开放水平要有新提升。加快构建开放型经济新体制，更好发挥《财富》全球论坛和世界华商大会后续效应，建立与国际惯例接轨的涉外管理和服务机制，营造国际化商务诚信和社会诚信环境，实际利用外资100亿美元以上。积极融入长江流域经济带和丝绸之路经济带，加强与沿海沿江沿边城市通关协作，依托航空、铁路口岸和综保区积极争取内陆自贸区，依托“蓉欧快铁”扩大蓉欧经贸合作，提升发展出口贸易和服务贸易，更好地服务企业“走出去”，完善72小时过境免签配套政策，办好2014中德对话论坛等国际交流活动，新增国际直飞航线3条，实现进出口总额500亿美元以上。更好发挥带头带动引领示范辐射作用，推动成都平原城市群建设，打造成都经济区一体化交通体系和金融信用体系，推进共建园区建设和区域公共服务、生态保护等合作。全面加强与长三角、珠三角、环渤海地区的经济合作。

四是民营经济发展要有新进展。加快投融资体制改革，实行统一的市场准入制度，建立负面清单管理模式，鼓励民间资本发展实体经济和参与城乡基础设施建设。创新和加大民营经济政策扶持力度，实施商事登记制度改革，优化民营企业诉求解决机制，形成各种市场主体平等参与市场竞争、同等受到法律保护的市场环境。创新中小企业融资机制，健全民营企业信用体系，完善中小微企业投融资平台，发展创业风险投资基金，发行小微企业成长保障债券，推动中小企业融资渠道多样化、融资成本合理化。鼓励发展混合所有制经济，健全国企法人治理结构，深化国企投融资改革，支持国有资本更多投向公益性基础设施和民生性公共服务。

二、推动城市空间转型升级，加快建设现代化大都市

坚持城乡一体、产城融合、四态合一，全面提升城乡规划建设水平，加快形成市域生产、生活、生态空间的合理结构，着力建设宜居宜业的现代化大都市。

一要优化市域城镇发展体系。完善市域城镇体系规划，完成卫星城发展规划修编，制定城镇及村庄规划技术管理规定，加快构建“一轴双核六走廊”、“多中心、组团式、网络化”的市域城镇空间结构。优化城市功能分区，规范中心城区空间格局，合理确定城市用地结构，统筹市域交通组织、生态绿地系统和地下空间利用，推动圈层融合发展，促进生产空间集约高效、生活空间宜居适度、生态空间山清水秀。优化城市风貌，实行中心城区建筑项目形态分级管理，深入挖掘历史文化底蕴，提升城市设计水平和人民南路、东郊记忆等重点区域形态，加快锦江水井坊等特色街区和金堂五凤镇等特色古镇建设。

二要加快推进天府新区建设。强化新区基础设施建设，加快地铁1号线南延线和“三纵一横”等重大交通项目建设，全线贯通天府大道南线，适度超前建设学校、医院等公共服务设施，同步规划建设电力、燃气、加油加气站和消防站，打造宜业宜商宜居现代化新城。加快新区产业发展，引进更多重大产业化项目，推进中国西部国际博览城、天府创新中心等重大项目建设，强化新川创新科技园、电子信息产业园、国际汽车城等功能区建设，加快建设天府创新科技城和秦皇寺中央商务区，促进现代制造业、高端服务业和都市现代农业加速聚集。提升新区生态环境，加快建设兴隆湖、鹿溪河湿地和锦江生态带等生态项目，加强河湖、交通干线绿化等生态建设，营造更加宜居环境。

三要大力推进交通主枢纽建设。加快对外通道建设，大力推进成蒲铁路、成兰铁路、火车北站改造等重大铁路项目建设，完成成渝客专土建工程，成灌快铁彭州支线、成绵乐城际铁路、第二绕城高速和成安渝高速建成通车。加快市域交通网络建设，推动绕城高速向城市道路转化，加强光华大道绕城互通等重要节点立交化改造、断头路改造和人行天桥建设，开通运行地铁2号线东延线，加快建设地铁3号线等3条在建线路，开工建设地铁1号线三期、5号线、10号线等5条线路，启动建设ITS综合交通信息服务平台。优化加密公交线网，提高公交覆盖面和便捷性，进一步提升公交出行分担率，实现中心城区和近郊公交一体化发展。大力建设“光网城市”和“无线城市”，加快推进4G应用，建成国家级互联网骨干直联点城市。

三、推动城市产业转型升级，加快构建现代产业体系

坚持发展高端产业和产业高端，突出重大项目，壮大实体经济，着力构建具有全球竞争力的现代产业体系。

一要大力发展先进制造业。强化重点产业发展，突出发展电子信息、汽车、石化和轨道交通产业，加快发展航空航天、生物医药、新能源、新材料和节能环保产业，优化发展冶金、食品、建材和轻工产业，大力培育新的工业增长点。强化重大项目促建，推动沃尔沃等项目量产达产，银鹭食品基地等项目竣工投产，确保重大项目完成投资500亿元以上。强化产业园区建设，做强做优近郊工业园区，加快发展远郊工业园区，进一步提升园区承载能力和服务功能。深入推进节能减排，加快淘汰落后产能，推动重点企业节能管理和技术改造。进一步加大龙头企业、骨干企业、小巨人企业和科技型企业培育力度，新增2户以上百亿企业。规模以上工业增加值增长12%。

二要提升发展现代服务业。深化现代服务业综合改革试点，扎实推进服务业核心城市建设。加快服务业差异化转型升级，推动现代技术与服务业融合发展，大力发展以大数据、云计算为核心的信息服务、电子商务、文化创意等新兴服务业，优先发展与先进制造业紧密关联的现代金融、现代商贸物流、会展服务、科技服务、商务服务等生产性服务业，促进旅游、餐饮、健康服务等生活性服务业转型升级。提升服务业发展业态，大力发展总部经济，推动楼宇经济提质增效，打造全产业链的国际国内货物贸易服务平台，强化现代服务业聚集区建设，推进高端服务业集群集约发展。服务业增加值增长9%。

三要积极发展都市现代农业。加强“米袋子”和“菜篮子”建设，划定并加大对粮田和菜田的政策支持，完善农产品价格风险防范体系和流通体系，保障主要农产品供给，新增益民菜市（店）20家以上。推动农业规模化高端化发展，深化“成新蒲”示范带和粮经产业新村综合示范基地建设，发展绿色农业、有机农业等高端农业和特色林业，建成农业示范园区150个、基地260个。健全农业社会化服务体系，启动建设智能化农业基地20个，综合机械化率达67%以上。加强农田水利建设，深入推进水库、渠系治理和建设，建成高标准农田10万亩，新增节水灌面13.3万亩。农业增加值增长3.3%。

四、推动城市生态转型升级，加快改善城乡人居环境

坚持生态文明，推进绿色发展，加大城乡环境治理力度，实行更严格的生态保护，不断提升人居环境质量。

一要狠抓大气污染整治。全面实施改善空气质量“十项措施”，从严治理涉气企业污染，加强餐饮企业油烟治理，完成中心城区企业清洁能源改造，全面实施火电、水泥、玻璃、化工等重点企业在线监控，坚持不达标就停产的刚性要求。强化机动车尾气治理，加强机动车排气污染检测和“黄标车”限行执法，全面供应新标准车用燃油。强化扬尘治理，严格建筑、市政、交通工地扬尘管理，加大街道除尘保洁力度，加强秸秆禁烧联防联控，提高秸秆综合利用率。提升大气污染防治能力，建立成都大气污染源清单，建成空气质量预测预报平台，提高重污染天气的应对能力。

二要深入推进水环境治理。启动第二批黑臭河渠治理，集中整治违法排污企业，加快市域污水管网建设，深入开展下河排水口污水综合治理，建成新建污水处理厂二期。加强河渠沿线水域环境治理，启动金马河综合整治，修复南河、西河、湔江等重要河流生态，加快实现河渠及沿岸无垃圾堆放、无淤泥堆积、无杂物漂浮、无违规建设。强化农村面源污染治理，完善乡镇污水处理厂配套管网，推广农村沼气池和配方施肥，深化农家乐、畜禽养殖场、屠宰场等废水治理。加强固体废弃物综合利用，强化饮用水水源地和地下水资源保护，加快李家岩第二水源建设前期工作。

三要全面加强城乡生态建设。划定全市生态保护红线，加强中心城区绿地系统建设，优化城市公园布局，增加“邮票绿地”和“袖珍广场”，加快打造骨干绿道，大力发展绿色建筑，中心城区新增公共绿地10万平方米。加快环城生态区建设，实施绕城生态带复耕植绿，推进金沙湖、江安湖等建设，建成北湖水生作物示范区、白鹭湾二期等湿地，加快构建“六湖八湿地”生态体系。打造城镇生态隔离区，发展城市走廊间组团式绿地，强化优质农田和水源保护，提升重点河道景观，启动全国水生态文明试点城市建设。保护提升龙门山、龙泉山生态屏障，巩固天然林保护、退耕还林等生态工程成果，建立健全生态补偿机制，强化生态保育、生物多样性保护和水源涵养。

五、推动城市管理转型升级，加快提升城市品质

坚持科学化民主化治理，构建城市综合管理体系，完善多元社会治理结构，确保城市高效运行、社会和谐稳定。

一要强化城市综合管理。毫不放松城乡环境综合治理，加大垃圾乱扔、摊位乱摆、车辆乱停等“七乱”现象整治力度，开展中心城区生活垃圾转运站点专项整治，强化违法广告、违章建筑等集中治理，加强城郊结合部、车站、市场等复杂区域环境综合治理，强化运渣车等特种车辆运营管理，提升城区重要节点路段美化水平。加强市容环境精细化管理，提升数字化城管水平，健全城市综合管理技术规范和业务标准，强化桥梁、井盖等市政设施管护，推进生活垃圾分类投放、不落地收运，加快万兴环保发电厂等环卫设施建设。完善城市管理机制，深化城市综合管理体制改革，探索建立大城管综合执法机制，启动市容秩序分类管理试点，推进市政设施维修维护保养作业市场化运行。

二要创新和加强社会治理。加快社会组织管理改革，稳妥推进社会组织直接登记和异地商会登记管理，重点培育和优先发展行业协会商会类、科技类、公益慈善类、城乡社区服务类等社会组织，推进政府向社会组织购买服务，鼓励社会组织参与社会治理。提升流动人口服务管理，强化流动人口信息分析预测与综合运用，探索流动人口积分制，实现流动人口服务管理向实有人口服务管理转变。提升社区自治水平，建立议事会成员联系村（居）民制度，推动社区、社会组织、社工人才队伍互动发展，完善社区用房等便民设施。深化信访制度改革，强化网上信访，推进涉法涉诉诉访分离。

三要强化法治成都建设。加强社会治安综合治理，强化网格化布警和动态化巡控，加强和创新虚拟社会管理，构建信息化立体化社会治安防控体系，深入实施社会治安专项行动，依法严厉打击各类违法犯罪活动，维护社会治安稳定。强化食品药品安全监管，完善四级食品药品监管体系和生产经营准入制度，提高食品药品检验检测能力，推进食品生产销售全程可追溯，保持打击食品药品违法犯罪行为高压态势，切实消除城郊结合部、校园、中药材等重点区域和领域安全隐患，争创首批国家食品安全示范城市。从严抓好安全生产，落实安全生产监管责任和主体责任，深化道路交通、消防、危化品、建筑工地、特种设备等重点行业领域安全生产日常监管，健全隐患排查治理和安全预防控制体系。深化全国法治城市创建，深入开展“六五”普法，健全社区矫正工作体系。加强重大事件应急管理，强化风险评估和舆情研判，加大重点领域和区域防控力度，加强人民调解、行政调解与司法调解的有机衔接，健全有效预防和化解社会矛盾机制，加快构建公共突发事件预防预警和应急处置体系，切实维护社会和谐稳定。进一步加强民族宗教工作。巩固和发展军政军民团结。

六、推动城乡形态转型升级，加快新型城镇化建设

坚持以人为核心，以新型工业化为动力，推进以工促农、以城带乡，促进现代城市和现代农村和谐相融、协调发展。

一要加快“北改”和棚户区改造。大力推进城北改造，加快打通城北出蓉大通道，强化重要连接道路、节点建设和断头路改造，加快茶花、火车北站、二仙桥等片区改造，启动云内动力等片区改造，健全医院、学校等配套设施，提升城北片区面貌。大力推进棚户区分类改造，规范房屋征收和补偿标准，强化政策统筹和市级支持力度，引导和鼓励信贷资金、民间资本参与改造，加快中心城区和县城老旧小区、厂房和市场改造。完善中心城区基础配套，加快公益性菜市场、停车场、变电站等公建配套建设，健全供排水、输气管网、电力等基础设施，新增100个公建配套设施，建成自来水七厂一期。

二要提升大中小城市和特色镇建设水平。加快现代化卫星城和区域中心城建设，完善与中心城区的快速通道，提升产城融合、职住平衡发展水平，推动龙泉驿、双流、郫县、新津、新（都）青（白江）、温江、都江堰等7个卫星城加快建成大城市，推动金堂等6个区域中心城加快建成中等城市，更好地疏解中心城区人口和功能。加快现代化小城市建设，完善与卫星城和区域中心城的一体化交通联系，健全公共服务，强化产业支撑，创新行政管理体制，增强综合承载能力，加强新繁、淮口、安德等小城市试点示范建设。加快特色镇建设，结合禀赋优势明确发展定位，加强产业分类指导，探索整镇推进新型城镇化，推动丽春、成佳、福洪等特色镇提升发展。深入推进一般场镇改造，深化乡镇重大建设项目民主管理改革，鼓励因地制宜发展传统特色产业，整体提升场镇面貌和发展动力。

三要大力推进美丽乡村建设。加快新农村综合体建设，按照小规模、组团式、生态化要求，依托交通走廊、河流和农业产业化项目，集中连片推进新农村综合体建设，新建和提升新农村综合体340个。深化村级公共服务和社会管理改革，优化村级专项资金使用方式，引导更多社会资本参与农村道路、饮水、能源、信息化等设施建设。提升农村基础设施水平，启动第二批农村公共服务和社会管理设施标准化建设，推广农村小型公共基础设施村民自建改革，加强老旧村庄综合改造和村落、村道环境整治，改建农村公路1500公里，基本解决农村人口饮水安全问题。

七、强化民生保障和改善，着力增进城乡居民福祉

坚持发展成果更多更公平惠及全体市民，加快社会事业改革，提升公共服务水平，更好地满足市民需求。

一要积极促进就业创业。实施更加积极的就业政策，推进离校未就业高校毕业生就业促进计划，强化农村转移劳动力、城镇困难人员等重点人群就业促进，建立经济发展与扩大就业联动机制，结合产业升级开发更多就业岗位。完善创业扶持政策，加大创业融资政策支持力度，健全创业服务体制机制，用好大学生创业园等创业载体，深入推进以创业带动就业。提升城乡就业服务水平，加快构建劳动者终身职业培训体系，健全失业动态监测预警和就业形势分析制度，优化就业服务管理信息系统，深化劳动保障监察“两网化”管理，加快新人才市场建设。稳妥推进收入分配制度改革。

二要着力提升社会保障水平。建立更加公平可持续的社保制度，健全多缴多得激励机制，实行城乡居民养老保险市级统筹，实现城镇职工、城乡居民养老保险无障碍转移接续，强化非公经济从业人员参保工作，推进城镇职工基本医保个人账户改革，深化医保付费方式和工伤保险改革，推广城乡基本医疗保险门诊特殊疾病管理。完善多元养老服务体系，启动第二批公建民营养老机构建设，鼓励支持民建民营养老机构建设，发展村（社区）居家养老服务中心，新增筹建养老床位7000张。健全覆盖城乡的社会救助体系，推动全市低保标准分圈层统一，健全留守儿童、妇女、残疾人、困境未成年人等重点人群权益保障体系，加强惠民殡葬工作。提升应保尽保的城乡住房保障水平，加快保障性安居工程建设，竣工保障性住房2万套，健全保障房小区常态化管理机制。

三要大力发展教育文化卫生事业。深化部省市共建教育改革试验区，启动新一轮学前教育提升工程，扩大公办和公益性幼儿园覆盖面，缓解“入园难”和“入园贵”问题，完成中小学标准化提升工程，开展中小学教育质量综合改革，加快发展特殊教育、继续教育和社区教育，完善现代职业教育体系，加速教育现代化国际化进程。提升公共文化服务水平，推动基层文化阵地提档升级和公共文化数字化建设，扎实推进文化惠民工程，深入开展“成都文化四季风”、全民阅读、文艺志愿服务、城乡文艺巡演等基层文化服务活动和“中国梦”等文艺主题创作活动，推进成都图书馆新馆、民办博物馆聚集中心、巴金文化博物馆等重大文化项目建设，加强文化遗产保护与利用。深化医药卫生体制改革，深入推进“医药分开”，改革药品供应保障制度，推广全科医生契约服务模式，支持公立医院建立现代医院管理制度。建立计生特殊家庭及精神病人家庭关爱扶助长效机制，稳妥实施“单独两孩”政策。深入开展“运动成都”等全民健身活动，推动公益性体育场馆免费开放，办好第一届世界太极拳锦标赛等国际赛事。

八、强化政府职能转变，着力建设法治政府和服务型政府

坚持依法行政、科学行政、廉洁行政，深化行政管理体制改革，创新行政管理方式，增强政府公信力和执行力。

一要严格依法行政。严格执行市人大决议决定，自觉接受市人大及其常委会的法律监督、工作监督和市政协的民主监督，健全人大代表、政协委员建议批评“绿色通道”，广泛听取民主党派、工商联、无党派人士和工会、共青团、妇联等人民团体意见，高度重视社会监督和舆论监督。健全规范性文件、重大决策合法性审查机制，完善法律顾问制度。进一步规范行政执法程序，全面落实行政执法责任制，加大行政执法监督力度，实施重大行政处罚和行政强制备案制度，健全行政复议机制。

二要推进科学行政。健全政府议事规则，完善重大事项公众参与、专家论证、风险评估、合法性审查和集体讨论相结合的决策制度。大力推进简政放权，规范精简行政审批事项，创新重大招商引资项目联动审批和全程代办服务机制，优化建设项目和市场准入并联审批流程，稳步推进事业单位分类改革。加强行政权力公开透明运行，提高重大决策公众参与度和重点领域信息公开度，整合市级政务信息平台，拓展电子政务大厅功能，加强政务微博等平台建设，畅通政民政企联系渠道。推进财税体制改革，建立全程预算绩效管理制度，加强各区（市）县政府性债务管理，严格落实新增债务预警控制机制，着力防控债务风险。

三要强化勤政廉政。巩固深化党的群众路线教育实践活动成果，深入开展维护群众权益九大专项行动，做好结对帮扶工作，切实帮助基层和群众排忧解难。深入推进廉洁成都建设，严格落实党风廉政建设责任制，健全惩治和预防腐败体系，加大财政预决算、重大项目、民生资金、环境保护等审计力度，严格领导干部经济责任审计，加强公共资源使用、管理、交易的监督检查，健全国企日常监管和产权转让制度。加强节约型机关建设，严格落实《党政机关厉行节约反对浪费条例》和《党政机关国内公务接待管理规定》，切实加强“三公”经费、会议活动、办公用房等管理，进一步降低公务成本。

此外，我们将切实按照省委省政府和市委统一部署，扎实抓好灾后重建各项工作，完善邛崃“3+5”灾后重建镇村规划，坚持用统筹城乡的思路和办法高质量推进农房重建，加快交通、水电、水利等重建项目建设，提升发展旅游、现代农业等特色优势产业，推动受灾乡镇整体面貌大幅提升，努力打造重建精品和全省灾后重建典范。

各位代表，做好2014年工作，任务艰巨，责任重大。让我们紧密团结在以习近平同志为总书记的党中央周围，高举中国特色社会主义伟大旗帜，在省委省政府和市委坚强领导下，团结一心、锐意进取、扎实工作，奋力打造西部经济核心增长极，为加快建设现代化国际化大都市而努力奋斗！
